# Unbinned model-independent measurements with coherent admixtures of multibody neutral *D* meson decays

**DOI:** 10.1140/epjc/s10052-018-5599-1

**Published:** 2018-02-10

**Authors:** Anton Poluektov

**Affiliations:** Department of Physics, University of Warwick, Coventry, UK

## Abstract

Various studies of Standard Model parameters involve measuring the properties of a coherent admixture of ${D} ^0$ and ${\overline{D}{}} {}^0$ states. A typical example is the determination of the Unitarity Triangle angle $\gamma$ in the decays $B\rightarrow DK$, $D\rightarrow {{K} ^0_\mathrm{\scriptscriptstyle S}} {{\pi } ^+} {{\pi } ^-}$. A model-independent approach to perform this measurement is proposed that has superior statistical sensitivity than the well-established method involving binning of the $D\rightarrow {{K} ^0_\mathrm{\scriptscriptstyle S}} {{\pi } ^+} {{\pi } ^-}$ decay phase space. The technique employs Fourier analysis of the complex phase difference between ${D} ^0$ and ${\overline{D}{}} {}^0$ decay amplitudes and can easily be generalised to other similar measurements, such as studies of charm mixing or determination of the angle $\beta$ from ${{B} ^0} \rightarrow D h^0$ decays.

## Introduction

Precise measurements of $$C\!P$$ violation in decays of beauty hadrons is one of the key methods to search for effects of physics beyond the Standard Model. The phenomenon of $$C\!P$$ violation is described in the Standard Model (SM) by the Cabibbo–Kobayashi–Maskawa (CKM) mechanism [1, 2], where $$C\!P$$ violation enters as a complex phase in the unitary $$3\times 3$$ matrix (CKM matrix) describing transitions between quarks of the three generations due to charged-current weak interactions. A common representation of the CKM matrix is the Unitarity Triangle (UT), the sides and angles of which are experimentally observable parameters. The fundamental $$C\!P$$-violating phase, the angle $$\gamma $$ of the UT (also known in the literature as $$\phi _3$$), can be obtained with extremely low theoretical uncertainty [3] from tree-dominated $${b} $$ hadron decays and thus serves as a “standard candle” for searches of effects beyond the Standard Model in other heavy flavour processes.

Various techniques have been proposed to measure $$\gamma $$ experimentally in the decays of *B* mesons into final states with neutral *D* mesons [4–7]. The $$C\!P$$ violation in these decays is generated by interference of $${b} \rightarrow {c} $$ and $${b} \rightarrow {u} $$ quark level transitions once the neutral *D* meson is reconstructed in a final state accessible to both $${D} ^0$$ and $${\overline{D}{}} {}^0$$ decays. The neutral *D* meson in this case forms a coherent admixture of $${{D} ^0} $$ and $${{\overline{D}{}} {}^0} $$ states which is denoted here as *D*. One of the most sensitive techniques involves analysis of the Dalitz plot density of multibody *D* decays such as $$D\rightarrow {{K} ^0_\mathrm{\scriptscriptstyle S}} {{\pi } ^+} {{\pi } ^-} $$  [8, 9].

Two different techniques have been developed and implemented experimentally to extract $$\gamma $$ from $$B\rightarrow DK$$ decays using multibody *D* meson final states. One is model-dependent, with the complex amplitude of the *D* decay obtained by fitting the flavour-specific $${{D} ^0} $$ decay density to a model [10–16]. This technique offers optimal statistical precision since the fit can be performed in an unbinned fashion, however, it suffers from uncertainty, which is difficult to quantify, due to modelling of the $${{D} ^0} $$ amplitude. Another method is a binned model-independent approach, where information as regards the behaviour of the strong phase across the phase space of the $${D} ^0$$ decay is obtained from samples of quantum-correlated $${{D} ^0} {{\overline{D}{}} {}^0} $$ decays produced near kinematic threshold [8, 17–20].

In the model-independent technique, one needs to determine the relation between the decay densities of quantum-correlated ${{D} ^0} {{\overline{D}{}} {}^0}$ decays and *D* decays from $B\rightarrow DK$. This necessarily requires estimation of the decay density from scattered data, which is achieved by binning both decay densities. Each bin is assigned a number of parameters that characterise the averaged behaviour of the amplitude (its magnitude and phase) over the bin; these parameters are obtained by solving a system of equations that also includes the value of $\gamma$. In general, the binned approach reduces statistical sensitivity compared to the unbinned model-dependent technique, but the procedure is developed in such a way that it produces an unbiased measurement even in the case of a very rough binning.

In this paper, a method to extract $$\gamma $$ is proposed which does not involve binning and aims to combine the advantages of the model-dependent and model-independent approaches. Like the binned approach with optimal binning, it uses a construction inspired by a $${{D} ^0} $$ amplitude model, but provides an unbiased measurement even if the wrong model is used. It is shown to offer better statistical sensitivity than the binned approach. The method employs Fourier analysis of the distribution of the complex phase difference between the $${D} ^0$$ and $${\overline{D}{}} {}^0$$ amplitudes. The method is illustrated using the “golden” channel $$B\rightarrow DK$$ with subsequent $$D\rightarrow {{K} ^0_\mathrm{\scriptscriptstyle S}} {{\pi } ^+} {{\pi } ^-} $$ decay, but can easily be generalised to other cases of $$\gamma $$ determination where the binned model-independent technique is applicable: analyses using other three- or four-body $${{D} ^0} $$ decays [21–26], multibody *B* decays [27, 28] or analyses using correlated Dalitz plots of multibody *B*- and *D*-meson decays [29, 30].

Apart from measurements of $$\gamma $$, similar model-independent techniques, which employ interference between $${{D} ^0} $$ and $${{\overline{D}{}} {}^0} $$ amplitudes, have been developed for other kinds of measurements: studies of $$C\!P$$ violation and mixing parameters of $${{D} ^0} $$ mesons [31–33], measurements of the UT angle $$\beta $$ in $${{B} ^0} \rightarrow Dh^0$$ (where $$h^0$$ is a neutral light meson) and $${{B} ^0} \rightarrow D{{\pi } ^+} {{\pi } ^-} $$ decays [34, 35]. In all these cases, the technique proposed can be applied instead of the binned methods.

## Model-independent formalism with weight functions

In this section, the formalism for $\gamma$ measurement is recalled to introduce the notation, and the established model-independent technique is reformulated in slightly different terms. This allows a demonstration that the binned approach is not the only possible method to perform such a measurement.

Measurements of $\gamma$ based on $B\rightarrow DK$ processes use the fact that the decay involves the interference of tree-dominated ${b} \rightarrow {c}$ and ${b} \rightarrow {u}$ diagrams, which produce neutral *D* mesons with opposite flavours. In the case of ${{{B} ^+}} \rightarrow D{{K} ^+}$ decays followed by $D\rightarrow {{K} ^0_\mathrm{\scriptscriptstyle S}} {{\pi } ^+} {{\pi } ^-}$, the amplitude as a function of two variables of the *D* decay Dalitz plot, the squared invariant masses $m^2_+ \equiv m^2_{{{K} ^0_\mathrm{\scriptscriptstyle S}} {{\pi } ^+}}$ and $m^2_-\equiv m^2_{{{K} ^0_\mathrm{\scriptscriptstyle S}} {{\pi } ^-}}$, is expressed as

1 $$\begin{aligned} \overline{A}_{B}(m^2_+, m^2_-) = \overline{A}_D(m^2_+, m^2_-) + r_Be^{i(\delta _B+\gamma )} A_D(m^2_+, m^2_-), \end{aligned}$$

where the first term is due to $\bar{{b}}\rightarrow \bar{{c}}$ and the second due to $\bar{{b}}\rightarrow \bar{{u}}$ transition. Here $A_D(m^2_+, m^2_-)$ is the amplitude of the ${{D} ^0} \rightarrow {{K} ^0_\mathrm{\scriptscriptstyle S}} {{\pi } ^+} {{\pi } ^-}$ decay, $\overline{A}_D(m^2_+, m^2_-)$ is that, for the ${{\overline{D}{}} {}^0} \rightarrow {{K} ^0_\mathrm{\scriptscriptstyle S}} {{\pi } ^+} {{\pi } ^-}$ decay, $r_B$ is the relative magnitude of the two contributions and $\delta _B$ is the $C\!P$-conserving strong phase between them. The amplitude $A_B$ for the $C\!P$-conjugated decay ${{{B} ^-}} \rightarrow D{{K} ^-}$ can be obtained by replacing $\gamma \rightarrow -\gamma$ and swapping the *D* decay amplitudes: $\overline{A}_D\leftrightarrow A_D$. A simultaneous analysis of the two amplitudes $A_B$ and $\overline{A}_B$ provides information on the unknown parameters $\gamma$, $r_B$, and $\delta _B$.

Experimentally, one deals with probability densities rather than amplitudes. The decay density for ${{{B} ^+}} \rightarrow D{{K} ^+}$ decays as a function of $\mathbf {z} \equiv (m^2_+, m^2_-)$ is

2 $$\begin{aligned}&\bar{p}_{{B}}(\mathbf {z}) \propto |\overline{A}_D(\mathbf {z}) + r_B e^{i\delta _B + i\gamma } A_D(\mathbf {z})|^2 \nonumber \\&\quad = |\overline{A}_D(\mathbf {z}) + (x_+ + iy_+) A_D(\mathbf {z})|^2, \end{aligned}$$

where the Cartesian $C\!P$-violating observables are introduced: $x_+ = r_B\cos (\delta _B+\gamma )$ and $y_+=r_B\sin (\delta _B+\gamma )$. The decay density $p_B(\mathbf {z})$ for ${{{B} ^-}} \rightarrow D{{K} ^-}$ decay involves the corresponding parameters $x_- = r_B\cos (\delta _B-\gamma )$ and $y_- = r_B\sin (\delta _B-\gamma )$:

3 $$\begin{aligned}&p_{{B}}(\mathbf {z}) \propto |A_D(\mathbf {z}) + r_B e^{i\delta _B - i\gamma } \overline{A}_{D}(\mathbf {z})|^2 \nonumber \\&\quad = |A_D(\mathbf {z}) + (x_- + iy_-) \overline{A}_D(\mathbf {z})|^2. \end{aligned}$$

The expressions for the decay densities can be rewritten as

4 $$\begin{aligned} \begin{aligned} \bar{p}_{{B}}(\mathbf {z})&= \bar{h}_{{B}}\left\{ \bar{p}_D(\mathbf {z}) + r_B^2 p_D(\mathbf {z}) + 2[x_+C(\mathbf {z}) - y_+S(\mathbf {z})]\right\} , \\ p_{{B}}(\mathbf {z})&= h_{{B}}\left\{ p_D(\mathbf {z}) + r_B^2 \bar{p}_D(\mathbf {z}) + 2[x_-C(\mathbf {z}) + y_-S(\mathbf {z})]\right\} , \end{aligned} \end{aligned}$$

where $h_{{B}}$ and $\bar{h}_{{B}}$ are the normalisation factors, and $p_D(\mathbf {z})$ and $\bar{p}_D(\mathbf {z})$ are the Dalitz plot densities of flavour-tagged ${{D} ^0} \rightarrow {{K} ^0_\mathrm{\scriptscriptstyle S}} {{\pi } ^+} {{\pi } ^-}$ and ${{\overline{D}{}} {}^0} \rightarrow {{K} ^0_\mathrm{\scriptscriptstyle S}} {{\pi } ^+} {{\pi } ^-}$ decays:

5 $$\begin{aligned} p_D(\mathbf {z})= & {} |A_D(\mathbf {z})|^2 = p_D(m^2_+, m^2_-), \end{aligned}$$

6 $$\begin{aligned} \bar{p}_D(\mathbf {z})= & {} |\overline{A}_D(\mathbf {z})|^2 = p_D(m^2_-, m^2_+), \end{aligned}$$

*i.e.* the Dalitz plot distributions for $${{D} ^0} $$ and $${{\overline{D}{}} {}^0} $$ decays are symmetric under the exchange $$m^2_+\leftrightarrow m^2_-$$ assuming $$C\!P$$ conservation in $${{D} ^0} $$ decays.^1^ The functions $$C(\mathbf {z})$$ and $$S(\mathbf {z})$$ contain information as regards the motion of the complex strong phase over the Dalitz plot which cannot be obtained from flavour-specific *D* meson decays:

7 $$\begin{aligned} C(\mathbf {z}) = \text{ Re }\left[ A_D^*(\mathbf {z})\overline{A}_D(\mathbf {z})\right] , \;\;\; S(\mathbf {z}) = \text{ Im }\left[ A_D^*(\mathbf {z})\overline{A}_D(\mathbf {z})\right] . \end{aligned}$$

One needs to know them to obtain the values of $C\!P$ violating parameters $x_{\pm }$ and $y_{\pm }$ from $\bar{p}_{{B}}(\mathbf {z})$ and $p_{{B}}(\mathbf {z})$.

In the model-dependent approach to measure $\gamma$, the strong phase motion is fixed by an amplitude model. The model-independent technique employs pairs of neutral *D* mesons produced at the kinematic threshold in the $e^+e^-\rightarrow {{D} ^0} {{\overline{D}{}} {}^0}$ process to obtain this information. In this case, the two *D* mesons are produced in a *P*-wave such that their wave function is antisymmetric. As a result, if both *D* mesons are reconstructed in the ${{K} ^0_\mathrm{\scriptscriptstyle S}} {{\pi } ^+} {{\pi } ^-}$ final state, the densities of two Dalitz plots will be correlated:

8 $$\begin{aligned} p_{DD}(\mathbf {z} _1, \mathbf {z} _2)= & {} \frac{1}{2}|A_D(\mathbf {z} _1)\overline{A}_D(\mathbf {z} _2) - A_D(\mathbf {z} _2)\overline{A}_D(\mathbf {z} _1)|^2\nonumber \\= & {} h_{DD}\left\{ p_D(\mathbf {z} _1)\bar{p}_D(\mathbf {z} _2) + p_D(\mathbf {z} _2)\bar{p}_D(\mathbf {z} _1) \right. \nonumber \\&\left. - 2 \left[ C(\mathbf {z} _1) C(\mathbf {z} _2) + S(\mathbf {z} _1)S(\mathbf {z} _2) \right] \right\} . \end{aligned}$$

Here the indices “1” and “2” correspond to the two decaying *D* mesons and $h_{DD}$ is a normalisation factor. The necessary information as regards $C(\mathbf {z})$ and $S(\mathbf {z})$ is present in Eq. (8), but it is not straightforward to obtain the explicit expressions for the functions $C(\mathbf {z})$ and $S(\mathbf {z})$ from the observable distributions $p_D(\mathbf {z})$, $\bar{p}_{D}(\mathbf {z})$ and $p_{DD}(\mathbf {z} _1, \mathbf {z} _2)$.

Equation (8) contains an ambiguity: it is invariant under rotation of the pair $C(\mathbf {z})$, $S(\mathbf {z})$ by an arbitrary phase $\Delta$:

9 $$\begin{aligned} \left( \begin{array}{c}C(\mathbf {z}) \\ S(\mathbf {z})\end{array}\right) \rightarrow \left( \begin{array}{rr}\cos \Delta &{} \sin \Delta \\ -\sin \Delta &{} \cos \Delta \end{array}\right) \left( \begin{array}{c}C(\mathbf {z}) \\ S(\mathbf {z})\end{array}\right) . \end{aligned}$$

This does not constitute a significant problem since it effectively results in the redefinition of the strong phase $\delta _B$, leaving the $C\!P$-violating phase $\gamma$ unaffected. The other abiguity is the change of sign of $C(\mathbf {z})$ or $S(\mathbf {z})$, which results in the change of sign for $\gamma$. Other decays of *D* mesons from correlated ${{D} ^0} {{\overline{D}{}} {}^0}$ pairs can offer additional information to resolve these ambiguities. For instance, decays where one of the *D* mesons is reconstructed in a $C\!P$ eigenstate and the other is reconstructed as ${{K} ^0_\mathrm{\scriptscriptstyle S}} {{\pi } ^+} {{\pi } ^-}$ constrain $C(\mathbf {z})$, and they resolve the ambiguity (9), as well as fix the sign for $C(\mathbf {z})$. The remaining ambiguity, the sign of $S(\mathbf {z})$, can be resolved by a weak model assumption using isobar parametrisation of the *D* decay amplitude [18]. In practice, several *D* decay modes are combined to measure the same strong phase parameters [36], but the description below will concentrate only on ${{D} ^0} {{\overline{D}{}} {}^0}$ pairs where both *D* mesons are decaying to ${{K} ^0_\mathrm{\scriptscriptstyle S}} {{\pi } ^+} {{\pi } ^-}$.

The model-independent technique can be built based on the observation that explicit expressions for the functions $C(\mathbf {z})$ and $S(\mathbf {z})$ are not needed to obtain $x_{\pm },y_{\pm }$. One can derive a number of independent equations from the expressions (8) and (4) by integrating both the right and the left parts of the equations multiplied by certain weight functions $w_n(\mathcal {D})$ from a family of functions indexed by $1\le n\le M$. Equation (8) then becomes

10 $$\begin{aligned} p_{DD,mn}\equiv & {} \int \limits _{\mathcal {D} _1,\mathcal {D} _2}w_m(\mathbf {z} _1)w_n(\mathbf {z} _2)p_{DD}(\mathbf {z} _1, \mathbf {z} _2)d\mathbf {z} _1 d\mathbf {z} _2 \nonumber \\= & {} h_{DD}\left\{ p_m \bar{p}_n + \bar{p}_m p_n - 2[C_m C_n + S_m S_n] \right\} , \qquad \end{aligned}$$

while Eq. (4) become

11 $$\begin{aligned} \bar{p}_{{B},n}\equiv & {} \int \limits _{\mathcal {D}}w_n(\mathbf {z}) \bar{p}_{{B}}(\mathbf {z}) d\mathbf {z} \nonumber \\= & {} \bar{h}_{{B}}\left\{ \bar{p}_n + r_B^2 p_n + 2[x_+C_n - y_+S_n] \right\} , \nonumber \\ p_{{B},n}\equiv & {} \int \limits _{\mathcal {D}}w_n(\mathbf {z}) p_{B}(\mathbf {z}) d\mathbf {z} \nonumber \\= & {} h_{{B}}\left\{ p_n + r_B^2 \bar{p}_n + 2[x_-C_n + y_-S_n] \right\} , \end{aligned}$$

where

12 $$\begin{aligned} p_n = \int \limits _{\mathcal {D}}w_n(\mathbf {z}) p_D(\mathbf {z}) d\mathbf {z} \;\;\;\; \bar{p}_n = \int \limits _{\mathcal {D}}w_n(\mathbf {z}) \bar{p}_D(\mathbf {z}) d\mathbf {z} \end{aligned}$$

and

13 $$\begin{aligned} C_n = \int \limits _{\mathcal {D}}w_n(\mathbf {z}) C(\mathbf {z}) d\mathbf {z}, \;\;\;\; S_n = \int \limits _{\mathcal {D}}w_n(\mathbf {z}) S(\mathbf {z}) d\mathbf {z}. \end{aligned}$$

The integration in Eqs. (11)–(13) is performed over the entire Dalitz plot $\mathcal {D}$ of the *D* decay, while for Eq. (10) double integral is performed over the Dalitz plots $\mathcal {D} _1$ and $\mathcal {D} _2$ of two decaying *D* mesons. Unlike in the binned formalism described in Refs. [17, 18], here the terms proportional to $|A_{D}(\mathbf {z})|\cdot |\overline{A}_{D}(\mathbf {z})|$ are not factored out, thus capital letters are used to distinguish the expressions of Eq. (13) from $c_i$ and $s_i$ coefficients commonly used in the binned formalism.

The values of weighted integrals for the flavour-specific *D* sample ($p_n$ and $\bar{p}_n$), *B* sample ($\bar{p}_{{B},n}$ and $p_{{B},n}$) and correlated ${{D} ^0} {{\overline{D}{}} {}^0}$ sample ($p_{DD,mn}$) can be obtained directly from each of the corresponding scattered data samples by replacing the integrals with sums over individual observed events. The values of the weighted integrals for the phase terms $C_n$ and $S_n$ are considered as free parameters constrained by Eq. (10). This allows the values of $x_{\pm }$ and $y_{\pm }$ to be obtained by solving the system of Eq. (10) and (11).

The family of weight functions $w_n$ can be chosen arbitrarily, but the performance of the method with a limited data sample will depend on this choice. The binned model-independent approach is a particular case of the considered formalism where the weight functions are of the form

14 $$\begin{aligned} w_n(\mathbf {z}) = \left\{ \begin{array}{l} 1 \quad \text{ if } \;\mathbf {z} \in \mathcal {D} _n,\\ 0 \quad \text{ otherwise. } \end{array}\right. \end{aligned}$$

Here $\mathcal {D} _n$ are non-overlapping regions of the Dalitz plot which define the bins.

To reach optimal statistical sensitivity, the binning has to be chosen in such a way as to maximise the interference term in Eq. (11). A good approximation to the optimum is known to be the binning based on the strong phase difference between the favoured and suppressed *D* decay amplitudes [18]. Specifically, if one defines the phase difference $\Phi (m^2_+, m^2_-)$ as

15 $$\begin{aligned} \Phi (m^2_+, m^2_-)= & {} \arg A^\mathrm{(model)}_D(m^2_+, m^2_-)\nonumber \\&-\arg A^\mathrm{(model)}_D(m^2_-, m^2_+), \end{aligned}$$

then the bin $\mathcal {D} _n$ ($1\le n\le M$) is the region of the phase space which satisfies

16 $$\begin{aligned} 2\pi (n-1/2)/M< & {} \Phi (m^2_+, m^2_-) \nonumber \\< & {} 2\pi (n+1/2)/M; \;\; m^2_+<m^2_-. \end{aligned}$$

The bins in the region with $m^2_+ > m^2_-$ are defined symmetrically with respect to exchange $m^2_+ \leftrightarrow m^2_-$ and have indices $n<0$. Here $A^\mathrm{(model)}_{D}(\mathbf {z})$ is an amplitude model that ideally should approach the true amplitude $A_D(\mathbf {z})$ to reach optimal statistical precision, but does not need to match it exactly to provide an unbiased measurement.

The following section shows how to construct an unbinned model-independent formalism using a model-based phase-difference function $\Phi (m^2_+, m^2_-)$ which will be a generalisation of the technique with phase-difference binning. For reasons which will become obvious, this approach will not be optimal from the point of view of statistical uncertainty, and it will serve solely as a demonstration. Subsequently, a more optimal approach based on a similar construction will be presented.

## Unbinned technique using Fourier series expansion of phase difference

Let $\Phi (\mathbf {z})\equiv \Phi (m^2_+, m^2_-)$ be the function defined by Eq. (15) that maps two-dimensional Dalitz plot coordinates $\mathbf {z}$ to the one-dimensional space represented by a phase difference $\phi$ between the ${\overline{D}{}} {}^0$ and ${D} ^0$ amplitudes at the same Dalitz plot point. One can now define probability densities as functions of $\phi = \Phi (\mathbf {z})$. The density of the flavour-specific *D* decay becomes

17 $$\begin{aligned} p_D(\phi ) = \int \limits _{\Phi (\mathbf {z})=\phi }p_D(\mathbf {z}) d\mathbf {z}. \end{aligned}$$

From the experimentalist’s point of view, this function is the probability density (PDF) of the $\Phi (\mathbf {z})$ value for a sample of flavour-specific $D\rightarrow {{K} ^0_\mathrm{\scriptscriptstyle S}} {{\pi } ^+} {{\pi } ^-}$ decays, and is a continuous generalisation of the number of events $K_n$ that enter the $n^\mathrm{th}$ bin in the approach with binning based on equal phase difference [18]. Following Eqs. (6) and (15), the density for the $C\!P$-conjugate decay is

18 $$\begin{aligned} \bar{p}_{D}(\phi )=p_D(-\phi ). \end{aligned}$$

After a similar mapping is applied to the correlated densities of the two $D\rightarrow {{K} ^0_\mathrm{\scriptscriptstyle S}} {{\pi } ^+} {{\pi } ^-}$ Dalitz plots of the ${{D} ^0} {{\overline{D}{}} {}^0}$ sample (8), the following PDF of the variables $\phi _1 = \Phi (\mathbf {z} _1)$ and $\phi _2 = \Phi (\mathbf {z} _2)$ is obtained:

19 $$\begin{aligned} p_{DD}(\phi _1, \phi _2)= & {} h_{DD}\left\{ p_D(\phi _1) \bar{p}_D(\phi _2) + \bar{p}_D(\phi _1) p_D(\phi _2)\right. \nonumber \\&\left. -2\left[ C(\phi _1)C(\phi _2) + S(\phi _1)S(\phi _2)\right] \right\} , \end{aligned}$$

where

20 $$\begin{aligned} C(\phi ) = \int \limits _{\Phi (\mathbf {z})=\phi }C(\mathbf {z})d\mathbf {z}, \;\;\; S(\phi ) = \int \limits _{\Phi (\mathbf {z})=\phi }S(\mathbf {z})d\mathbf {z}. \;\;\; \end{aligned}$$

From the definitions (20) and (15) it follows that $C(\phi )$ is an even function, while $S(\phi )$ is odd:

21 $$\begin{aligned} C(-\phi )=C(\phi ),\;\;\;S(-\phi ) = -S(\phi ). \end{aligned}$$

Switching to the phase-difference representation for the $B^{\pm }\rightarrow DK^{\pm }$ densities (4), one obtains

22 $$\begin{aligned} \begin{aligned} \bar{p}_{{B}}(\phi ) = \bar{h}_{{B}}\left\{ \bar{p}_D(\phi ) + r_B^2 p_D(\phi ) + 2[x_+ C(\phi ) - y_+ S(\phi )]\right\} ,\\ p_{{B}}(\phi ) = h_{{B}}\left\{ p_D(\phi ) + r_B^2 \bar{p}_D(\phi ) + 2[x_- C(\phi ) + y_- S(\phi )]\right\} . \end{aligned} \end{aligned}$$

The next step is to choose the family of weight functions to construct a system of equations which allow the determination of $x_{\pm }$ and $y_{\pm }$ from Eqs. (19) and (22). Since the densities as functions of $\phi$ are periodic by construction, it appears that the natural choice is to use Fourier expansion of the functions of the phase difference, *i.e.* use weight functions of the form $\cos (n\phi )$ and $\sin (n\phi )$, where *n* is an integer number. The unknowns $x_{\pm }$ and $y_{\pm }$ will then enter the system of equations which relates the coefficients of the Fourier expansions of the $p_D$, $p_{DD}$, $\bar{p}_{{B}}$ and $p_{{B}}$ densities.

Specifically, the functions $p_D(\phi )$, $C(\phi )$ and $S(\phi )$ can be represented as

23 $$\begin{aligned} p_D(\phi )= & {} \frac{a^D_0}{2} + \sum \limits _{n=1}^{M} [ a^D_n \cos (n\phi ) + b^D_n \sin (n\phi ) ], \end{aligned}$$

24 $$\begin{aligned} \bar{p}_D(\phi )= & {} \frac{a^D_0}{2} + \sum \limits _{n=1}^{M} [ a^D_n \cos (n\phi ) - b^D_n \sin (n\phi ) ], \end{aligned}$$

25 $$\begin{aligned} C(\phi )= & {} \frac{a^C_0}{2} + \sum \limits _{n=1}^{M} a^C_n \cos (n\phi ), \end{aligned}$$

26 $$\begin{aligned} S(\phi )= & {} \sum \limits _{n=1}^{M} b^S_n \sin (n\phi ), \end{aligned}$$

keeping in mind that $C(\phi )$ is even and $S(\phi )$ is odd. The two-dimensional density $p_{DD}$ is represented by the four sets of Fourier coefficients $a^{DD}_{nm}$, $b^{DD}_{nm}$, $c^{DD}_{nm}$, and $d^{DD}_{nm}$, defined as

27 $$\begin{aligned} p_{DD}(\phi _1, \phi _2)= & {} \frac{a^{DD}_{00}}{4} + \sum \limits _{m=1}^{M}\frac{a^{DD}_{m0}}{2}\cos (m\phi _1) \nonumber \\&+ \sum \limits _{n=1}^{M}\frac{a^{DD}_{0n}}{2}\cos (n\phi _2) + \nonumber \\&\times \sum \limits _{n=1}^{M}\frac{b^{DD}_{0n}}{2}\sin (n\phi _2) + \sum \limits _{m=1}^{M}\frac{c^{DD}_{m0}}{2}\sin (m\phi _1) + \nonumber \\&\times \sum \limits _{m,n=1}^{M}[ a^{DD}_{mn}\cos (m\phi _1)\cos (n\phi _2) \nonumber \\&+\,b^{DD}_{mn}\cos (m\phi _1)\sin (n\phi _2) \nonumber \\&+ c^{DD}_{mn}\sin (m\phi _1)\cos (n\phi _2) \nonumber \\&+\, d^{DD}_{mn}\sin (m\phi _1)\sin (n\phi _2) ]. \end{aligned}$$

Strictly speaking, the equations above are exact only in the limit $M\rightarrow \infty$; however, in practice one has to truncate the Fourier series at a certain finite *M*.

For $p_D(\phi )$, the values of the Fourier coefficients can be calculated directly from scattered data $\phi ^{(i)}$, $i=1\ldots N_D$:

28 $$\begin{aligned} a^D_n = \frac{1}{\pi }\sum \limits _{i=1}^{N_D}\cos (n\phi ^{(i)}), \;\; b^D_n = \frac{1}{\pi }\sum \limits _{i=1}^{N_D}\sin (n\phi ^{(i)}), \end{aligned}$$

where $N_D$ is the number of events in the data sample and $\phi ^{(i)}=\Phi (\mathbf {z} ^{(i)})$ are the calculated phase-difference values for the data sample entries $\mathbf {z} ^{(i)}$. Similarly, the coefficients of the Fourier expansion for the correlated ${{D} ^0} {{\overline{D}{}} {}^0}$ sample can be calculated from the 2D scattered data $\phi ^{(i)}_1=\Phi (\mathbf {z} ^{(i)}_1), \phi ^{(i)}_2=\Phi (\mathbf {z} ^{(i)}_2)$, $i=1\ldots N_{DD}$ as

29 $$\begin{aligned} \begin{aligned} a^{DD}_{mn}&= \frac{1}{\pi }\sum \limits _{i=1}^{N_{DD}}\cos (m\phi _1^{(i)})\cos (n\phi _2^{(i)}), \;\; \\ b^{DD}_{mn}&= \frac{1}{\pi }\sum \limits _{i=1}^{N_{DD}}\cos (m\phi _1^{(i)})\sin (n\phi _2^{(i)}), \\ c^{DD}_{mn}&= \frac{1}{\pi }\sum \limits _{i=1}^{N_{DD}}\sin (m\phi _1^{(i)})\cos (n\phi _2^{(i)}), \;\; \\ d^{DD}_{mn}&= \frac{1}{\pi }\sum \limits _{i=1}^{N_{DD}}\sin (m\phi _1^{(i)})\sin (n\phi _2^{(i)}). \\ \end{aligned} \end{aligned}$$

On the other hand, from Eq. (19) one can obtain a set of relations between the Fourier coefficients for flavour-specific and ${{D} ^0} {{\overline{D}{}} {}^0}$ densities:

30 $$\begin{aligned} \begin{aligned} a^{DD}_{mn}&= 2 h_{DD}\left( a^D_m a^D_n - a^C_m a^C_n\right) , \\ b^{DD}_{mn}&= c^{DD}_{mn} = 0, \\ d^{DD}_{mn}&= -2 h_{DD}\left( b^D_m b^D_n + b^S_m b^S_n\right) . \\ \end{aligned} \end{aligned}$$

Equations (30) can be used to obtain the unknown coefficients $a^C_n$ and $b^S_n$ from the known values of $(a,b)^{D}_n$ and $(a,b,c,d)^{DD}_{mn}$. The system of Eq. (30) is solvable for any $M\ge 1$ (there are $2M^2+M+1$ independent equations and $2M+2$ unknown parameters). In practice, since the system of equations is overconstrained for $M>1$, it should be solved using a maximum likelihood fit, which will also provide estimate of the covariance matrix.

A maximum likelihood fit needs uncertainties for the coefficients that enter the equations. These can be calculated analytically by applying a Poisson bootstrapping technique [37]. Each term entering the sum in Eq. (28) or (29) is multiplied by a random number which follows the Poisson distribution with unit mean value. The variances for the sums can then be obtained assuming they have a Gaussian distribution (which is a valid assumption for large $N_D$):

31 $$\begin{aligned} \sigma ^2(a^D_n) = \frac{1}{\pi }\sum \limits _{i=1}^{N_D}\cos ^2(n\phi ^{(i)}), \;\; \sigma ^2(b^D_n) = \frac{1}{\pi }\sum \limits _{i=1}^{N_D}\sin ^2(n\phi ^{(i)}), \end{aligned}$$

and

32 $$\begin{aligned} \begin{aligned} \sigma ^2(a^{DD}_{mn})&= \frac{1}{\pi }\sum \limits _{i=1}^{N_{DD}}\cos ^2(m\phi _1^{(i)})\cos ^2(n\phi _2^{(i)}), \;\; \\ \sigma ^2(b^{DD}_{mn})&= \frac{1}{\pi }\sum \limits _{i=1}^{N_{DD}}\cos ^2(m\phi _1^{(i)})\sin ^2(n\phi _2^{(i)}), \\ \sigma ^2(c^{DD}_{mn})&= \frac{1}{\pi }\sum \limits _{i=1}^{N_{DD}}\sin ^2(m\phi _1^{(i)})\cos ^2(n\phi _2^{(i)}), \;\; \\ \sigma ^2(d^{DD}_{mn})&= \frac{1}{\pi }\sum \limits _{i=1}^{N_{DD}}\sin ^2(m\phi _1^{(i)})\sin ^2(n\phi _2^{(i)}). \\ \end{aligned} \end{aligned}$$

In addition, unlike in the binned case where the yields in each of the bins are statistically independent, the coefficients of the Fourier series are in general correlated. The covariance matrix can be calculated similarly using Poisson bootstrapping, *e.g.* the covariance between the $a_n$ and $b_m$ coefficients can be calculated as

33 $$\begin{aligned} \mathrm{cov}(a^D_n, b^D_m) = \frac{1}{\pi }\sum \limits _{i=1}^{N_D}\cos (n\phi ^{(i)})\sin (m\phi ^{(i)}). \end{aligned}$$

Similarly, the expressions for covariances between $a_n$ and $a_m$, $b_n$ and $b_m$, or between the coefficients $(a,b,c,d)^{DD}_{mn}$ can be obtained.

**Fig. 1:**
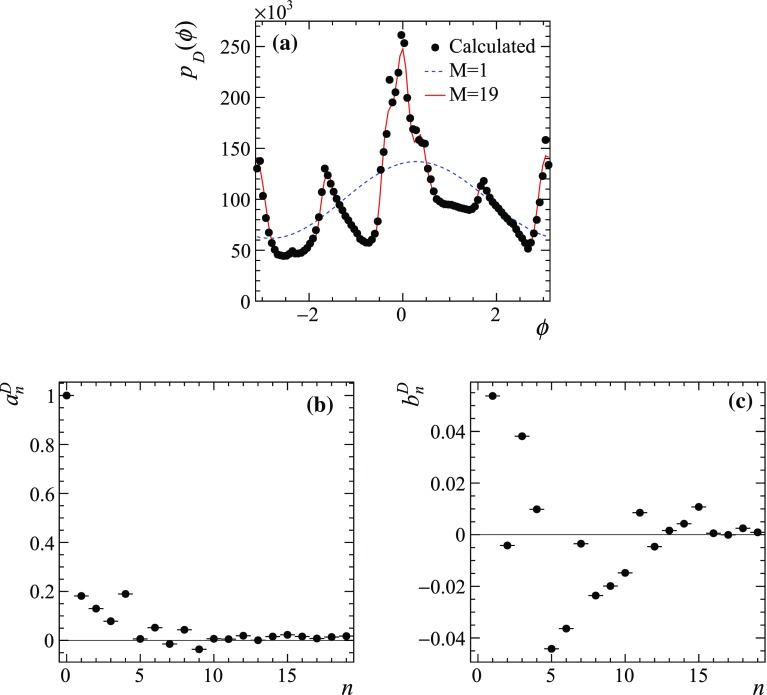
**a** The function $p_D(\phi )$ according to a model obtained by the Belle collaboration [14]. The points are the histogram of $\phi =\Phi (\mathbf {z})$ values calculated for the generated sample of flavour-specific $D\rightarrow {{K} ^0_\mathrm{\scriptscriptstyle S}} {{\pi } ^+} {{\pi } ^-}$ decays, solid red line is the result of Fourier expansion with $M=19$, and dashed blue line is a single harmonic ($M=1$). **b**, **c** Fourier series coefficients, calculated from the $p_D(\phi )$ density

Once the coefficients $(a,b)^{C,S}_n$ are obtained, they can be used to constrain the values of $x_{\pm }, y_{\pm }$ (and thus $\gamma$). Taking Fourier expansions of the functions $\bar{p}_{{B}}(\mathbf {z})$ and $p_{{B}}(\mathbf {z})$

34 $$\begin{aligned} \begin{aligned} \bar{p}_{{B}}(\phi )&= \frac{\bar{a}^B_0}{2} + \sum \limits _{n=1}^{M} [ \bar{a}^B_n \cos (n\phi ) + \bar{b}^B_n \sin (n\phi ) ], \\ p_{{B}}(\phi )&= \frac{ a^B_0}{2} + \sum \limits _{n=1}^{M} [ a^B_n \cos (n\phi ) + b^B_n \sin (n\phi ) ], \end{aligned} \end{aligned}$$

and plugging them into Eq. (22), one obtains the following system of equations:

35 $$\begin{aligned} \begin{aligned} \bar{a}^B_n&= \bar{h}_B\left[ \,\,\,\,(1 + r_B^2) a^D_n + 2x_+ a^C_n\right] , \\ \bar{b}^B_n&= \bar{h}_B\left[ -(1 - r_B^2) b^D_n - 2y_+ b^S_n\right] . \\ a^B_n&= h_B\left[ \,\,\,\,(1 + r_B^2) a^D_n + 2x_- a^C_n\right] , \\ b^B_n&= h_B\left[ \,\,\,\,(1 - r_B^2) b^D_n + 2y_- b^S_n\right] , \\ \end{aligned} \end{aligned}$$

which can be solved again using a maximum likelihood fit for any $M\ge 1$, after the extraction of the coefficients $(\bar{a},\bar{b})^B_n$ and $(a,b)^B_n$ and their uncertainties and correlations from the $B\rightarrow DK$ sample in a similar way. Alternatively, both sets of equations (30) and (35) can be solved simultaneously using a single combined likelihood.

**Fig. 2:**
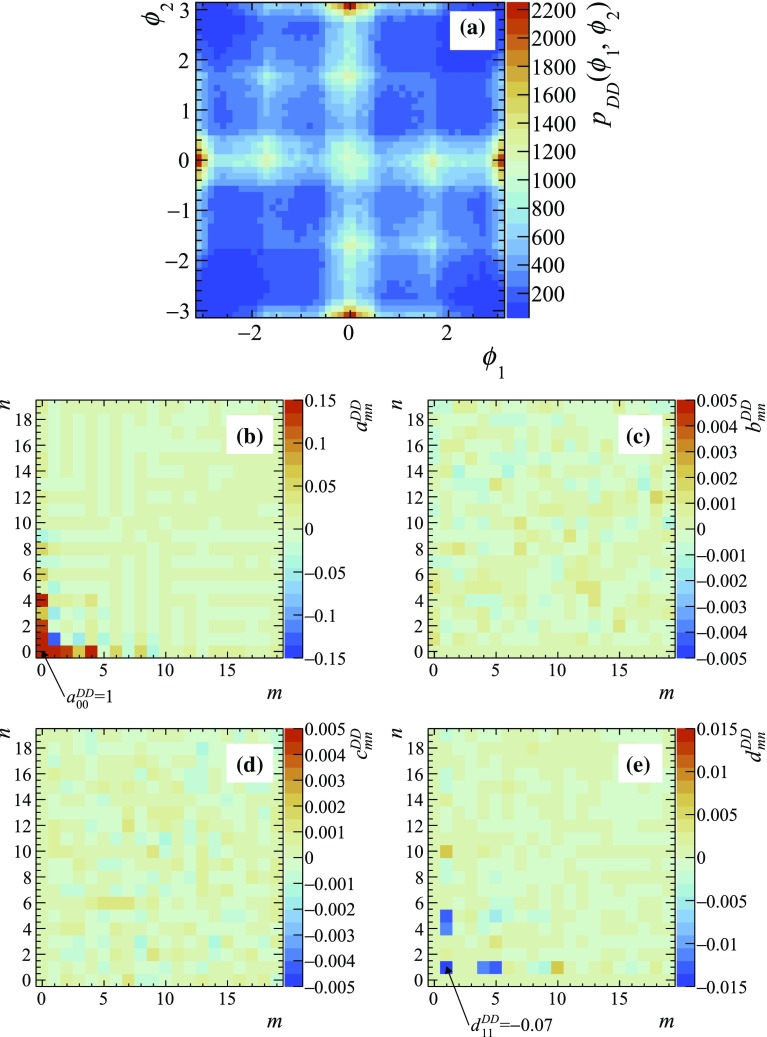
**a** The function $p_{DD}(\phi _1, \phi _2)$ according to a model obtained by the Belle collaboration [14], and **b**–**e** its Fourier series coefficients. The ranges of the plots for $a^{DD}_{mn}$ and $d^{DD}_{mn}$ are chosen such that the dominant components $a^{DD}_{00}=1$ and $d^{DD}_{11}=-0.07$ fall out of the range, to make the other components visible

**Fig. 3:**
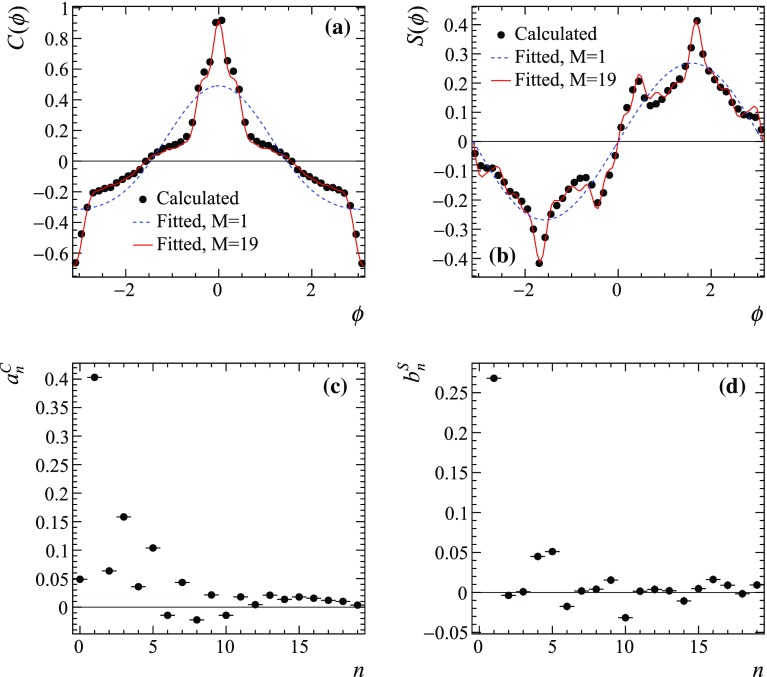
**a** The functions $C(\phi )$ and **b** $S(\phi )$ according to a model obtained by the Belle collaboration [14]. The points are the functions obtained from the true amplitude $A_D(\mathbf {z})$, solid red line is the function reconstructed from Fourier expansion with $M=19$, and dashed blue line is a single harmonic ($M=1$). **c**, **d** Fourier series coefficients for $C(\phi )$ and $S(\phi )$, respectively

As an illustration, the functions $p_D(\phi )$, $p_{DD}(\phi _1,\phi _2)$, $C(\phi )$ and $S(\phi )$ obtained using the $D\rightarrow {{K} ^0_\mathrm{\scriptscriptstyle S}} {{\pi } ^+} {{\pi } ^-}$ amplitude model $A_D(m^2_+, m^2_-)$ from the Belle measurement [14] and their respective coefficients of the Fourier expansion are shown in Figs. 1, 2 and 3. The function $p_D(\phi )$ shown in Fig. 1a is obtained by plotting the distribution of the function $\phi =\Phi (\mathbf {z})$ (black points) for events generated according to PDF $p(\mathbf {z})$. Its Fourier coefficients $a^D_n$ and $b^D_n$ up to $n=19$ are shown in Fig 1b, c, respectively. Since the normalisation is arbitrary, the coefficients are normalised such that $a^{D}_0=1$. The solid red line in Fig. 1a shows the result of Fourier expansion up to $n=19$, and the dashed blue line shows the first harmonic (expansion up to $n=1$). In the $p_{DD}(\phi )$ function that is obtained similarly by plotting the two-dimensional distribution of $\phi _1=\Phi (\mathbf {z} _1), \phi _2=\Phi (\mathbf {z} _2)$ for the correlated Dalitz plot points generated according to the $p_{DD}(\mathbf {z} _1, \mathbf {z} _2)$ density (Fig. 2), only the $a^{DD}_{mn}$ and $d^{DD}_{mn}$ coefficients are non-zero, while $b^{DD}_{mn}$ and $c^{DD}_{mn}$ are consistent with zero as expected from Eq. (30). The normalisation $a^{DD}_{00}=1$ is used.

The true functions $C(\phi )$ and $S(\phi )$ can be obtained, on one hand, from the known amplitude $A_D(\mathbf {z})$ by plotting the distribution of the function $\phi =\Phi (\mathbf {z})$ for events generated uniformly across the Dalitz plot with event-by-event weights $\sqrt{p_D(\mathbf {z}) \bar{p}_D(\mathbf {z})}\cos \Phi (\mathbf {z})$ and $\sqrt{p_D(\mathbf {z}) \bar{p}_D(\mathbf {z})}\sin \Phi (\mathbf {z})$, respectively. These functions are shown in Fig. 3a, b as black points. On the other hand, the functions can be reconstructed from the spectral coefficients $a^C_n$ and $b^S_n$ obtained from Eq. (30). The fitted coefficients $a^{C}_n$ and $b^{S}_n$ are plotted in Fig. 3c, d, while the functions $C(\phi )$ and $S(\phi )$ reconstructed from them are shown in Fig. 3a, b as solid red lines (from the coefficients up to $n=19$) and dashed blue line (only one harmonic, $n=1$). It can be seen from Fig. 3c, d that the highest “power” of the $C(\phi )$ and $S(\phi )$ spectrum is contained in the first harmonic, $n=1$. As a result, as will be seen from further studies with pseudoexperiments, limiting $M=1$ is sufficient to reach good sensitivity to $\gamma$.

## Strategy with Fourier expansion on split Dalitz plot

The strategy outlined above is the simplest example of the approach using Fourier expansion of the phase-difference distribution to measure $\gamma$. However, it is clear that this approach is not optimal from the point of view of statistical precision. The reason is that one integrates over all points of the phase space with the same expected phase difference, regardless of the magnitudes of the interfering ${{D} ^0} \rightarrow {{K} ^0_\mathrm{\scriptscriptstyle S}} {{\pi } ^+} {{\pi } ^-}$ and ${{\overline{D}{}} {}^0} \rightarrow {{K} ^0_\mathrm{\scriptscriptstyle S}} {{\pi } ^+} {{\pi } ^-}$ amplitudes. This effectively reduces the interference term and, as a consequence, the sensitivity to the relative phase between the two amplitudes. For similar reasons, the “optimal” binning scheme was introduced for the binned model-independent approach to improve the precision with the equal phase-difference binning [18].

The simplest way to improve the situation with the technique described here is to split the Dalitz plot into regions with comparable ratios between the absolute values of the interfering amplitudes, and to perform Fourier expansion in those regions separately. This approach is illustrated below in an example with the Dalitz plot split into two regions.

**Fig. 4:**
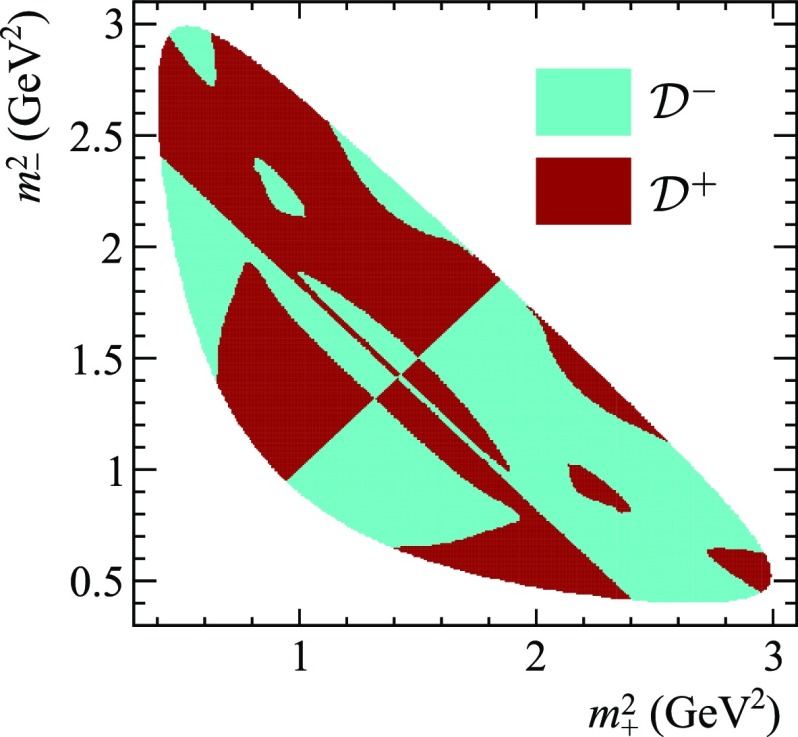
Splitting of $D\rightarrow {{K} ^0_\mathrm{\scriptscriptstyle S}} {{\pi } ^+} {{\pi } ^-}$ Dalitz plot into regions $\mathcal {D} ^+$ and $\mathcal {D} ^{-}$

Two regions of the Dalitz plot are considered: one with $\bar{p}_D(\mathbf {z})>p_{D}(\mathbf {z})$ (denoted region $\mathcal {D} ^+$) and the other with $\bar{p}_D(\mathbf {z})<p_{D}(\mathbf {z})$ (region $\mathcal {D} ^-$). These are shown in Fig. 4 for the same $D\rightarrow {{K} ^0_\mathrm{\scriptscriptstyle S}} {{\pi } ^+} {{\pi } ^-}$ amplitude model as used in the previous section. Clearly, the exchange $m^2_+ \leftrightarrow m^2_-$ transforms $\mathcal {D} ^+$ into $\mathcal {D} ^-$. Now one has to deal with two independent distributions for the $D\rightarrow {{K} ^0_\mathrm{\scriptscriptstyle S}} {{\pi } ^+} {{\pi } ^-}$ density, $p^+_D$ and $p^-_D$, as functions of the phase difference $\phi$, defined as

36 $$\begin{aligned} p^{\pm }_D(\phi ) = \int \limits _{\Phi (\mathbf {z})=\phi ;\;\; \mathbf {z} \in \mathcal {D} ^{\pm }} p_D(\mathbf {z}) d\mathbf {z}. \end{aligned}$$

The corresponding distributions for the $C\!P$-conjugated decays are

37 $$\begin{aligned} \bar{p}^{\pm }_D(\phi ) = \int \limits _{\Phi (\mathbf {z})=\phi ;\;\; \mathbf {z} \in \mathcal {D} ^{\pm }} \bar{p}_D(\mathbf {z}) d\mathbf {z} = p^{\mp }_D(-\phi ). \end{aligned}$$

It should be stressed that the superscripts “$$+$$” and “−” denote two Dalitz plot regions rather than *B* meson flavours. Throughout this paper, the flavour ($${b} $$ or $$\bar{{b}}$$) is consistently denoted by the absence or presence of a “bar” in the corresponding quantities, for example $$\bar{p}_{{B}}$$ and $$p_B$$, except for the subscript for $$C\!P$$-violating parameters $$x_{\pm }, y_{\pm }$$, which is a commonly used notation.

With the Dalitz plot split in this way, one needs to define two sets of functions $C(\phi )$ and $S(\phi )$ in the two Dalitz plot regions:

38 $$\begin{aligned} C^{\pm }(\phi ) =\int \limits _{\Phi (\mathbf {z})=\phi ;\;\; \mathbf {z} \in \mathcal {D} ^{\pm }} C(\mathbf {z})d\mathbf {z}, \;\;\; S^{\pm }(\phi ) = \int \limits _{\Phi (\mathbf {z})=\phi ;\;\; \mathbf {z} \in \mathcal {D} ^{\pm }} S(\mathbf {z})d\mathbf {z}. \;\;\; \end{aligned}$$

These functions will not be even and odd, as in the previous example, but instead they will satisfy the following properties:

39 $$\begin{aligned} C^-(\phi ) = C^+(-\phi ), \;\;\; S^-(\phi ) = -S^+(-\phi ). \end{aligned}$$

The two-dimensional density of the ${{D} ^0} {{\overline{D}{}} {}^0}$ sample will be described by a set of four functions $p_{DD}^{++}$, $p_{DD}^{+-}$, $p_{DD}^{-+}$ and $p_{DD}^{--}$ defined as

40 $$\begin{aligned} p_{DD}^{s_1 s_2}(\phi _1, \phi _2) = \int \limits _{ \begin{array}{l}\scriptstyle \Phi (\mathbf {z} _1)=\phi _1;\;\; \mathbf {z} _1\in \mathcal {D} ^{s_1}; \\ \scriptstyle \Phi (\mathbf {z} _2)=\phi _2;\;\; \mathbf {z} _2\in \mathcal {D} ^{s_2}\end{array}} p_{DD}(\mathbf {z} _1, \mathbf {z} _2) d\mathbf {z} _1 d\mathbf {z} _2, \end{aligned}$$

where $$s_1, s_2=\{$$“$$+$$”, “−”$$\}$$.

The Fourier expansion coefficients $a^{D\pm }_n$ and $b^{D\pm }_n$ for the *D* decay densities $p^{\pm }_D(\phi )$ are defined as

41 $$\begin{aligned} p^{\pm }_D(\phi ) = \frac{a^{D\pm }_0}{2} + \sum \limits _{n=1}^{M} [ a^{D\pm }_n \cos (n\phi ) + b^{D\pm }_n \sin (n\phi ) ], \end{aligned}$$

and similar coefficients for $\bar{p}_D(\phi )$ are denoted $\bar{a}^{D\pm }_n$ and $\bar{b}^{D\pm }_n$. In the case of $C\!P$ conservation in *D* decay, following Eq. (37), they are related as

42 $$\begin{aligned} \bar{a}^{D\pm }_n = a^{D\mp }_n,\;\;\; \bar{b}^{D\pm }_n = -b^{D\mp }_n. \end{aligned}$$

The Fourier expansion coefficients $(a,b)^{C\pm }_n$ and $(a,b)^{S\pm }_n$ for the $C^{\pm }(\phi )$ and $S^{\pm }(\phi )$ functions, respectively, are defined as

43 $$\begin{aligned} C^{\pm }(\phi ) = \frac{a^{C\pm }_0}{2} + \sum \limits _{n=1}^{M}[a^{C\pm }_n \cos (n\phi ) + b^{C\pm }_n \sin (n\phi )] \end{aligned}$$

and

44 $$\begin{aligned} S^{\pm }(\phi ) = \frac{a^{S\pm }_0}{2} + \sum \limits _{n=1}^{M}[a^{S\pm }_n \cos (n\phi ) + b^{S\pm }_n \sin (n\phi )], \end{aligned}$$

and they are related as

45 $$\begin{aligned} a^{C-}_n = a^{C+}_n,\;\; b^{C-}_n=-b^{C+}_n,\;\;a^{S-}_n=-a^{S+}_n,\;\;b^{S-}_n=b^{S+}_n. \end{aligned}$$

The relations between the coefficients of the Fourier expansion of the ${{D} ^0} {{\overline{D}{}} {}^0}$ and flavour *D* decay densities in that case take the following form:

46 $$\begin{aligned} a^{DD++}_{mn}= & {} h_{DD}[a^{D+}_m \bar{a}^{D+}_n + \bar{a}^{D+}_m a^{D+}_n \nonumber \\&\quad - 2(a^{C+}_m a^{C+}_n + a^{S+}_m a^{S+}_n)], \nonumber \\ a^{DD-+}_{mn}= & {} h_{DD}[a^{D+}_m \bar{a}^{D-}_n + \bar{a}^{D+}_m a^{D-}_n \nonumber \\&\quad - 2(a^{C+}_m a^{C-}_n + a^{S+}_m a^{S-}_n)], \nonumber \\ a^{DD+-}_{mn}= & {} h_{DD}[a^{D-}_m \bar{a}^{D+}_n + \bar{a}^{D-}_m a^{D+}_n \nonumber \\&\quad - 2(a^{C-}_m a^{C+}_n + a^{S-}_m a^{S+}_n)], \nonumber \\ a^{DD--}_{mn}= & {} h_{DD}[a^{D-}_m \bar{a}^{D-}_n + \bar{a}^{D-}_m a^{D-}_n \nonumber \\&\quad - 2(a^{C-}_m a^{C-}_n + a^{S-}_m a^{S-}_n)], \nonumber \\ b^{DD++}_{mn}= & {} h_{DD}[a^{D+}_m \bar{b}^{D+}_n + \bar{a}^{D+}_m b^{D+}_n \nonumber \\&\quad - 2(a^{C+}_m b^{C+}_n + a^{S+}_m b^{S+}_n)], \nonumber \\ b^{DD-+}_{mn}= & {} h_{DD}[a^{D+}_m \bar{b}^{D-}_n + \bar{a}^{D+}_m b^{D-}_n \nonumber \\&\quad - 2(a^{C+}_m b^{C-}_n + a^{S+}_m b^{S-}_n)],\nonumber \\ b^{DD+-}_{mn}= & {} h_{DD}[a^{D-}_m \bar{b}^{D+}_n + \bar{a}^{D-}_m b^{D+}_n \nonumber \\&\quad - 2(a^{C-}_m b^{C+}_n + a^{S-}_m b^{S+}_n)], \nonumber \\ b^{DD--}_{mn}= & {} h_{DD}[a^{D-}_m \bar{b}^{D-}_n + \bar{a}^{D-}_m b^{D-}_n \nonumber \\&\quad - 2(a^{C-}_m b^{C-}_n + a^{S-}_m b^{S-}_n)], \nonumber \\ c^{DD++}_{mn}= & {} h_{DD}[b^{D+}_m \bar{a}^{D+}_n + \bar{b}^{D+}_m a^{D+}_n \nonumber \\&\quad - 2(b^{C+}_m a^{C+}_n + b^{S+}_m a^{S+}_n)], \nonumber \\ c^{DD-+}_{mn}= & {} h_{DD}[b^{D+}_m \bar{a}^{D-}_n + \bar{b}^{D+}_m a^{D-}_n \nonumber \\&\quad - 2(b^{C+}_m a^{C-}_n + b^{S+}_m a^{S-}_n)], \nonumber \\ c^{DD+-}_{mn}= & {} h_{DD}[b^{D-}_m \bar{a}^{D+}_n + \bar{b}^{D-}_m a^{D+}_n \nonumber \\&\quad - 2(b^{C-}_m a^{C+}_n + b^{S-}_m a^{S+}_n)], \nonumber \\ c^{DD--}_{mn}= & {} h_{DD}[b^{D-}_m \bar{a}^{D-}_n + \bar{b}^{D-}_m a^{D-}_n \nonumber \\&\quad - 2(b^{C-}_m a^{C-}_n + b^{S-}_m a^{S-}_n)], \nonumber \\ d^{DD++}_{mn}= & {} h_{DD}[b^{D+}_m \bar{b}^{D+}_n + \bar{b}^{D+}_m b^{D+}_n \nonumber \\&\quad - 2(b^{C+}_m b^{C+}_n + b^{S+}_m b^{S+}_n)], \nonumber \\ d^{DD-+}_{mn}= & {} h_{DD}[b^{D+}_m \bar{b}^{D-}_n + \bar{b}^{D+}_m b^{D-}_n \nonumber \\&\quad - 2(b^{C+}_m b^{C-}_n + b^{S+}_m b^{S-}_n)], \nonumber \\ d^{DD+-}_{mn}= & {} h_{DD}[b^{D-}_m \bar{b}^{D+}_n + \bar{b}^{D-}_m b^{D+}_n \nonumber \\&\quad - 2(b^{C-}_m b^{C+}_n + b^{S-}_m b^{S+}_n)], \nonumber \\ d^{DD--}_{mn}= & {} h_{DD}[b^{D-}_m \bar{b}^{D-}_n + \bar{b}^{D-}_m b^{D-}_n \nonumber \\&\quad - 2(b^{C-}_m b^{C-}_n + b^{S-}_m b^{S-}_n)], \end{aligned}$$

where the coefficients $(a,b,c,d)^{DDs_1 s_2}_{mn}$ are defined similarly to those in Eq. (27), and the two superscripts $s_1, s_2=\{$“$+$”, “−”$\}$ correspond to the superscripts of the $p_{DD}^{s_1 s_2}$ functions (40). The coefficients $(\bar{a},\bar{b})^{D\pm }$ and $(a,b)^{(C,S)-}$ can be substituted by $(a,b)^{D\pm }$ and $(a,b)^{(C,S)+}$, respectively, using Eqs. (42) and (45), reducing the number of free parameters to fit. This substitution is, however, not done in Eq. (46) to emphasise the symmetry of the equations.

Finally, the equations for the densities of the *D* decay from $B^{\pm }\rightarrow DK^{\pm }$ take the following form in the split Dalitz plot case:

47 $$\begin{aligned} \begin{aligned} \bar{a}^{B+}_n&= \bar{h}_B\left\{ \bar{a}^{D+}_n + r_B^2 a^{D+}_n + 2[x_+ a^{C+}_n - y_+ a^{S+}_n]\right\} , \\ \bar{a}^{B-}_n&= \bar{h}_B\left\{ \bar{a}^{D-}_n + r_B^2 a^{D-}_n + 2[x_+ a^{C-}_n + y_+ a^{S-}_n]\right\} , \\ \bar{b}^{B+}_n&= \bar{h}_B\left\{ \bar{b}^{D+}_n + r_B^2 b^{D+}_n + 2[x_+ b^{C+}_n - y_+ b^{S+}_n]\right\} , \\ \bar{b}^{B-}_n&= \bar{h}_B\left\{ \bar{b}^{D-}_n + r_B^2 b^{D-}_n + 2[x_+ b^{C-}_n + y_+ b^{S-}_n]\right\} , \\ a^{B+}_n&= h_B\left\{ a^{D+}_n + r_B^2 \bar{a}^{D+}_n + 2[x_- a^{C+}_n + y_- a^{S+}_n]\right\} , \\ a^{B-}_n&= h_B\left\{ a^{D-}_n + r_B^2 \bar{a}^{D-}_n + 2[x_- a^{C-}_n - y_- a^{S-}_n]\right\} , \\ b^{B+}_n&= h_B\left\{ b^{D+}_n + r_B^2 \bar{b}^{D+}_n + 2[x_- b^{C+}_n + y_- b^{S+}_n]\right\} , \\ b^{B-}_n&= h_B\left\{ b^{D-}_n + r_B^2 \bar{b}^{D-}_n + 2[x_- b^{C-}_n - y_- b^{S-}_n]\right\} . \\ \end{aligned} \end{aligned}$$

The number of unknown *D* phase parameters in the equations has now increased: there are $4M+2$ independent coefficients $(a,b)^{C,S+}_n$ ($0\le n\le M$ for *a* and $1\le n\le M$ for *b*) plus a common normalisation factor $h_{DD}$ in the system of equations (46). Nevertheless, the statistical precision in this approach appears to be better as will be seen in the feasibility study.

In principle, one can even consider splitting the Dalitz plot into more regions, but certainly the increase in the number of free parameters can diminish the possible gain in statistical precision. Any strategy involving splitting the Dalitz plot should be optimised taking into account the size of experimentally available samples of correlated ${{D} ^0} {{\overline{D}{}} {}^0}$ and $B\rightarrow DK$ decays.

## Simulation results

To test the feasibility of the proposed method, simulation studies using pseudoexperiments are performed. Samples of flavour-specific $D\rightarrow {{K} ^0_\mathrm{\scriptscriptstyle S}} {{\pi } ^+} {{\pi } ^-}$ decays, correlated ${{D} ^0} {{\overline{D}{}} {}^0}$ pairs decaying to ${{K} ^0_\mathrm{\scriptscriptstyle S}} {{\pi } ^+} {{\pi } ^-}$, and $D\rightarrow {{K} ^0_\mathrm{\scriptscriptstyle S}} {{\pi } ^+} {{\pi } ^-}$ decays from $B\rightarrow DK$ are generated using the *D* decay amplitude measured by Belle collaboration [14]. Samples are simulated with $r_B=0.1$, $\gamma =60^{\circ }$ and $\delta _B=130^{\circ }$, which is close to the results of the recent model-independent measurement of the $B\rightarrow DK$, $D\rightarrow {{K} ^0_\mathrm{\scriptscriptstyle S}} {{\pi } ^+} {{\pi } ^-}$ channel by the LHCb collaboration [19]. For each of those event samples, the Fourier series coefficients and their covariance matrices are calculated as described in Sect. 3. Systems of equations which contain relations between Fourier spectrum coefficients of flavour-specific *D*, ${{D} ^0} {{\overline{D}{}} {}^0}$ and $B\rightarrow DK$ densities are then solved by maximising the combined likelihood to obtain the value of $\gamma$.

The formalism in Sects. 3 and 4 involved Cartesian $C\!P$-violating parameters $x_{\pm }$ and $y_{\pm }$. This approach is likely more suitable when dealing with real data when one has to combine the results of different $\gamma$-sensitive analyses. In the simulation study presented here, the free parameters are chosen to be $\gamma$, $r_B$ and $\delta _B$.

For the flavour-specific $D\rightarrow {{K} ^0_\mathrm{\scriptscriptstyle S}} {{\pi } ^+} {{\pi } ^-}$ mode, a large sample of $10^7$ generated events is used. This sample is not expected to contribute significantly to the uncertainty on $\gamma$ since high-statistics data sets are available at both the *B* factories and the LHCb. The size of the $B\rightarrow DK$, $D\rightarrow {{K} ^0_\mathrm{\scriptscriptstyle S}} {{\pi } ^+} {{\pi } ^-}$ sample generated is $10^4$ events for each *B* meson flavour, which corresponds roughly to 10 times the data sample from LHCb Run 1 [19]. Three scenarios with different correlated ${{D} ^0} {{\overline{D}{}} {}^0}$ sample sizes are considered, $10^5$, $10^4$ and $10^3$ events. For comparison, the $e^+e^-\rightarrow {{D} ^0} {{\overline{D}{}} {}^0}$ data sample collected by CLEO experiment where both *D* mesons decay into ${{K} ^0_\mathrm{\scriptscriptstyle S}} {{\pi } ^+} {{\pi } ^-}$ contains 473 events, however, many other *D* decay modes are used in the combined fit to obtain the phase coefficients (notably, the modes where one of the *D* mesons is reconstructed in a $C\!P$ eigenstate or as ${{K} ^0_\mathrm{\scriptscriptstyle L}} {{\pi } ^+} {{\pi } ^-}$) [36]. It is expected that the statistical uncertainty of the ${{D} ^0} {{\overline{D}{}} {}^0}$ sample of $10^5$ events will contribute negligibly to the uncertainty on $\gamma$, thus pseudoexperiments with this sample size probe uniquely how the approximation of the amplitude with a finite number of parameters (*i.e.* truncated Fourier series or limited number of bins) affects $\gamma$ sensitivity. The low-statistics sample of $10^3$ events, on the other hand, will demonstrate the contribution of a limited ${{D} ^0} {{\overline{D}{}} {}^0}$ sample to the sensitivity.

Each ensemble of pseudoexperiments is fitted with the binned model-independent procedure with 3, 5, 8, 12, and 20 bins using both the phase-difference and the “optimal” binning schemes [18], and with the two Fourier analysis techniques outlined above, using the entire Dalitz plot or the Dalitz plot split in two regions, respectively. In the approaches with Fourier expansion, the limit *M* on the number of harmonics is set to $M=1, 2, 4, 7, 11$, or 19. In addition, an unbinned model-dependent fit is performed to serve as a reference for the best possible statistical $\gamma$ precision that can be reached.

**Fig. 5:**
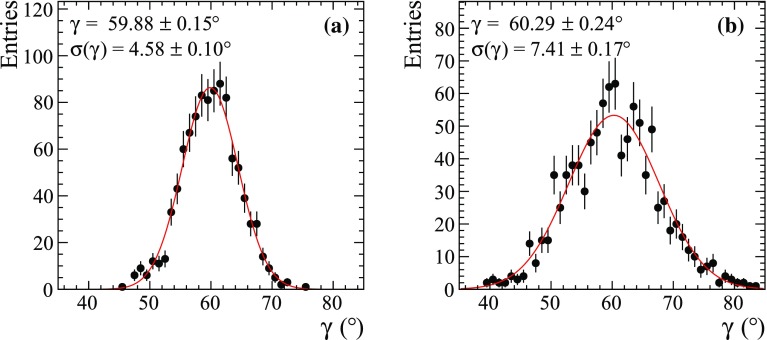
Distributions of the reconstructed value of $\gamma$ in pseudoexperiments with the **a** baseline and **b** reduced $D\rightarrow {{K} ^0_\mathrm{\scriptscriptstyle S}} {{\pi } ^+} {{\pi } ^-}$ model used for phase-difference calculation. Points are histograms of fit results, and the solid red line is the result of a fit with Gaussian distribution. The numerical results of the Gaussian fit are also reported

The Fourier expansion approach is verified to produce unbiased results if different $D\rightarrow {{K} ^0_\mathrm{\scriptscriptstyle S}} {{\pi } ^+} {{\pi } ^-}$ amplitudes are used for event generation and calculation of the phase difference $\Phi (\mathbf {z})$. This is certainly a requirement for a technique to be model-independent. This check is performed by using a reduced $D\rightarrow {{K} ^0_\mathrm{\scriptscriptstyle S}} {{\pi } ^+} {{\pi } ^-}$ model where a subset of two-body amplitudes is present ($\rho (770)^0$, $\omega (782)$ and $f_0(980)$ in the ${{\pi } ^+} {{\pi } ^-}$ amplitude, and $K^*(892)^{\pm }$ in the ${{K} ^0_\mathrm{\scriptscriptstyle S}} \pi$ amplitudes) plus a flat non-resonant term. The results in Fig. 5 are shown for $M=1$ and $10^5$ ${{D} ^0} {{\overline{D}{}} {}^0}$ events, but a similar check is performed for each value of *M*.

**Fig. 6:**
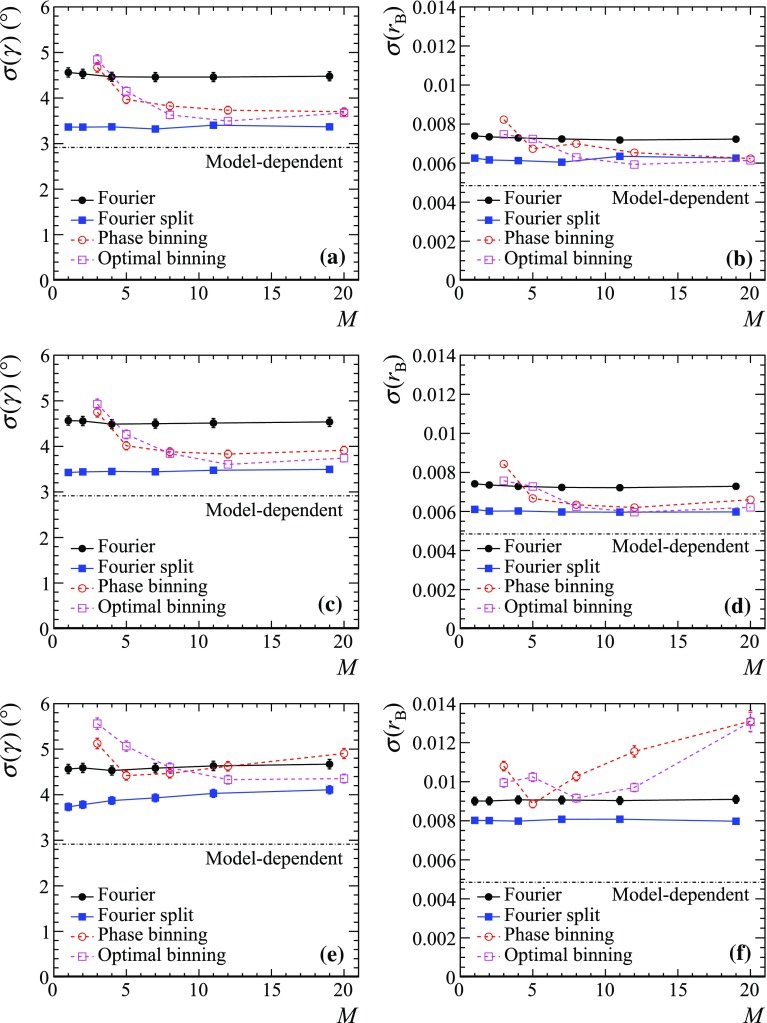
Statistical precision of **a**, **c**, **e** $\gamma$ and **b**, **d**, **f** $r_B$ measurement as a function of the number of Fourier terms (in the unbinned) or number of bins (in the binned approach) for different model-independent fit strategies, and its comparison with the model-dependent unbinned fit. The generated ${{D} ^0} {{\overline{D}{}} {}^0}$ sample size is **a**, **b** $10^5$, **c**, **d** $10^4$ and **e**, **f** $10^3$ events

Figure 6 shows the $\gamma$ and $r_B$ resolutions as functions of the number of bins (for the binned scenarios) and the number of Fourier expansion terms (for the unbinned scenarios) with the four fit strategies described above and for the three different ${{D} ^0} {{\overline{D}{}} {}^0}$ sample sizes. For comparison, the uncertainty of the unbinned model-dependent fit is also shown. While the precision of the binned approaches depends on the number of bins, the uncertainty of the Fourier expansion techniques practically does not depend on the number of harmonics *M* for relatively large ${{D} ^0} {{\overline{D}{}} {}^0}$ samples sizes, while for a small ${{D} ^0} {{\overline{D}{}} {}^0}$ sample size of $10^3$ the optimum is reached for $M=1$ (*i.e.* for the smallest possible number of free parameters, which is three for non-split and six for split Dalitz plot). It is possible that other multibody *D* decays may require higher harmonics to reach optimal sensitivity. Another case when Fourier terms with $n>1$ might be required is if the amplitude model $A_D^\mathrm{(model)}(\mathbf {z})$ used to define $\Phi (\mathbf {z})$ differs significantly from the true one.

**Table 1:** Uncertainty of $\gamma$ measurement with strategies using binned fit (with optimal binning) and using Fourier expansion (with non-split and split Dalitz plot)

Sample size	$\gamma$ resolution (${}^{\circ }$)		
	Binned optimal	Fourier non-split	Fourier split
$10^4$ $B\rightarrow DK$, $10^3$ ${{D} ^0} {{\overline{D}{}} {}^0}$	$4.33\pm 0.10$	$4.54\pm 0.10$	$3.73\pm 0.08$
$10^4$ $B\rightarrow DK$, $10^4$ ${{D} ^0} {{\overline{D}{}} {}^0}$	$3.60\pm 0.08$	$4.51\pm 0.10$	$3.43\pm 0.08$
$10^4$ $B\rightarrow DK$, $10^5$ ${{D} ^0} {{\overline{D}{}} {}^0}$	$3.49\pm 0.10$	$4.47\pm 0.10$	$3.32\pm 0.08$

The numbers correspond to the best $\gamma$ resolution obtained in a range of *M* (see Fig. 6). For comparison, the $\gamma$ uncertainty for unbinned model-dependent fit is $\sigma (\gamma )=2.91\pm 0.07^{\circ }$

The $\gamma$ uncertainties for the optimal scenarios with the binned and unbinned techniques are compared in Table 1. The uncertainty of the approach with split Dalitz plot is significantly better than when the Dalitz plot is taken as a whole. It is also clear that the Fourier expansion technique with split Dalitz plot shows better sensitivity than the binned method using “optimal” binning, with the gain being the most significant for smaller ${{D} ^0} {{\overline{D}{}} {}^0}$ sample size. The technique, however, is still about 10% less sensitive than the unbinned model-dependent approach. The possibilities to further improve the sensitivity of the unbinned model-independent method are discussed in Sect. 7.

## Practical considerations

To be applicable to real data, the technique should be able to deal with experimental effects such as backgrounds and non-uniform detection efficiency across the Dalitz plot. Since the background enters the decay density additively, it can be treated at the level of Fourier-transformed variables, by calculating the Fourier expansion of the background density and subtracting it from the coefficients calculated on data. On the other hand, the efficiency enters the density in a multiplicative way, thus Fourier expansion need to be applied to efficiency-corrected data. The correction can be applied on an event-by-event basis, by assigning each event a weight proportional to the inverse of efficiency while calculating the Fourier coefficients.

The studies presented above have been performed using a combined likelihood fit to both $B\rightarrow DK$ and correlated ${{D} ^0} {{\overline{D}{}} {}^0}$ samples. It is also possible to perform the analysis in two stages, by first calculating the coefficients of Fourier transformation of the functions $C(\phi )$ and $S(\phi )$ from the ${{D} ^0} {{\overline{D}{}} {}^0}$ data, followed by a fit to $B\rightarrow DK$ sample using the coefficients, their correlations and uncertainties from the first stage. This is likely to be more convenient in practice, since the data samples come from different experiments.

## Further directions of development

Using notation of the generalised model-independent formalism presented in Sect. 2, the Fourier analysis technique proposed above uses a family of $2M+1$ weight functions

48 $$\begin{aligned} \begin{aligned} w_{0}(\mathbf {z})&= 1, \\ w_{n}(\mathbf {z})&= \cos [n\Phi (\mathbf {z})], \\ w_{n+M}(\mathbf {z})&= \sin [n\Phi (\mathbf {z})], \\ \end{aligned} \end{aligned}$$

where $1\le n\le M$. The use of the function $\Phi (\mathbf {z})$ ensures that different points in the phase space do not cancel each other out while calculating the integral, and thus the interference term that provides sensitivity to $C\!P$-violating observables is large (assuming, of course, that the amplitude model that provides $\Phi (\mathbf {z})$ is close to the true amplitude). However, information about the absolute value of the amplitude is ignored in the formalism presented in Sect. 3 and is taken into account only rather roughly in Sect. 4. Alternatively, one could consider a weight function that in addition takes into account the magnitudes of the favoured and suppressed amplitudes from the model $|\overline{A}^\mathrm{(model)}_D(\mathbf {z})|$ and $|A^\mathrm{(model)}_D(\mathbf {z})|$, and thus adds more information to maximise the interference term. In the presence of background, the family of weight functions should also take into account the distribution of background events over the phase space. Further optimisation of the family of weight functions needs additional study.

The proposed technique could be especially useful in the cases where a binned approach will limit precision due to small sample sizes of decays which determine the phase information. Examples are the ${{D} ^0} \rightarrow {{K} ^0_\mathrm{\scriptscriptstyle S}} {{K} ^+} {{K} ^-}$ mode, where the sample of quantum-correlated decays is small and currently only two bins are used in the $\gamma$ measurement [19]. Another example is $B\rightarrow DK\pi$ decays, where the phase coefficients corresponding to the three-body *B* decay are free parameters together with $\gamma$ [29, 30]. Having an amplitude model which describes the strong phase variation across the *B* decay Dalitz plot with a small number of parameters should improve the statistical sensitivity.

Other analyses, where the coherent $${{D} ^0} $$–$${{\overline{D}{}} {}^0} $$ admixtures are involved, are measurements of charm mixing and $$C\!P$$ violation in mixing and measurement of the UT angle $$\beta $$ in $$B\rightarrow D h^0$$ decays. These classes of measurements utilise oscillations of $${{D} ^0} $$ and $${{B} ^0} $$ mesons, respectively, and thus the parameters of the $${{D} ^0} $$–$${{\overline{D}{}} {}^0} $$ admixture are functions of decay time. In the proposed formalism, the coefficients of the Fourier series will be functions of decay time as well. While such analyses will certainly be more complicated than the case with constant coefficients, they are conceptually similar to the measurements using the binned technique which have already been carried out [33, 35].

## Conclusion

A technique to perform unbinned model-independent analysis of a coherent admixture of ${D} ^0$ and ${\overline{D}{}} {}^0$ states decaying to a multibody final state is proposed. It is illustrated in detail using the measurement of the UT angle $\gamma$ from $B\rightarrow DK$ decays. Unlike the well-known technique with Dalitz plot binning, the proposed method employs Fourier analysis of the spectrum of the strong phase difference between the ${D} ^0$ and ${\overline{D}{}} {}^0$ amplitudes. While the method relies on an amplitude model to reach optimal statistical precision, it is unbiased by construction even if the wrong model is used.

A study of the feasibility of the proposed method has been performed with simulated pseudoexperiments. The precision of the method does not depend strongly on the number of Fourier expansion terms used, and even with only the single leading term yields sensitivity comparable to that of the binned model-independent approach. A modification of the procedure, where Fourier expansion is performed in two regions of the Dalitz plot separated according to the ratio of the suppressed and favoured amplitudes, provides $\gamma$ sensitivity better than the most optimal binned strategy. The gain compared to the binned approach is especially significant if the size of the correlated ${{D} ^0} {{\overline{D}{}} {}^0}$ sample, which determines the strong phase in *D* meson decay, is small. Possible ways of improving the sensitivity of the proposed technique even further are identified and need further study.

The method is not limited to $$\gamma $$ measurements with three-body *D* decays and can be generalised to any analysis where the parameters of a coherent admixture of $${{D} ^0} $$ and $${{\overline{D}{}} {}^0} $$ in a multibody final state need to be determined, such as measurements of charm mixing and $$C\!P$$ violation, and measurements of the UT angle $$\beta $$ in $$B\rightarrow D h^0$$ decays. The technique could also be useful in $$\gamma $$ measurements with a double Dalitz plot analysis of $$B\rightarrow DK\pi $$, $$D\rightarrow {{K} ^0_\mathrm{\scriptscriptstyle S}} {{\pi } ^+} {{\pi } ^-} $$ decay; in that case the Fourier expansion can be applied to both the *B* and the *D* Dalitz plots.

## Footnotes

1 The same assumption is made throughout this paper.
